# Impact of reflexive preoperative molecular testing for indeterminate nodules on lobectomy and completion thyroidectomy rates

**DOI:** 10.1530/ETJ-25-0189

**Published:** 2026-03-13

**Authors:** Caitlin T Yeo, Jiahui Wu, Sana Ghaznavi, Paul Stewardson, Samar Amanullah, Adrian Box, Markus Eszlinger, Ralf Paschke

**Affiliations:** ^1^ Department of Surgery, University of Calgary, Calgary, Canada; ^2^ Department of Oncology, University of Calgary, Calgary, Canada; ^3^ Department of Medical Science, University of Calgary, Calgary, Canada; ^4^ Department of Medicine, University of Calgary, Calgary, Canada; ^5^ Department of Pathology and Laboratory Medicine, University of Calgary, Calgary, Canada

**Keywords:** thyroid cancer, lobectomy, completion thyroidectomy, molecular testing, indeterminate nodules

## Abstract

**Objective:**

Guideline-based lobectomy criteria were adopted in July 2017, and reflexive molecular testing (MT) was introduced for all Bethesda III/IV indeterminate thyroid nodules (ITNs) in July 2020. This study evaluates how reflexive MT of ITNs influenced lobectomy and completion thyroidectomy (CT) rates.

**Methods:**

Patients with well-differentiated thyroid cancer who had undergone surgery were identified from a prospective database. Patients were categorized into three groups: i) Bethesda V/VI initial lobectomy, ii) Bethesda III/IV initial lobectomy without MT, iii) Bethesda III/IV lobectomy candidates with MT. The indications for and rates of lobectomy and CT, postoperative complications, and thyroid hormone replacement (THR) were assessed.

**Results:**

From July 2017 to October 2023, 227 of 799 (28%) patients with thyroid cancer underwent initial lobectomy, with 91, 70, and 64 patients in each group, respectively. Included in group 3 were 16 ITNs that would have undergone diagnostic lobectomy; however, due to the presence of a malignant mutation, they underwent total thyroidectomy (TT). TT was the appropriate surgery for 15 of 16 patients based on either preoperative lobectomy exclusion criteria or final pathology. CT rates by group were 26, 43, and 27%, respectively (*P* < 0.05). Vascular invasion, tumor size >4 cm, and aggressive histology were the most common indications for CT. ThyroSPEC was independently associated with a lower likelihood of CT in multivariate analysis. Surgical complications were lower with lobectomy (2%) versus CT (7%), with 44% of lobectomy patients requiring THR.

**Conclusion:**

Reflexive MT for ITNs decreased CT rates by increasing appropriate upfront TT. Lobectomy demonstrated lower rates of surgical complications and THR than two-stage thyroidectomy.

## Introduction

In 2015, the American Thyroid Association (ATA) published thyroid lobectomy recommendations for indeterminate and malignant thyroid nodules (1). Thyroid lobectomy is considered an appropriate definitive treatment option for low-recurrence-risk intrathyroidal papillary thyroid cancers (PTCs), reflecting an improved understanding of their indolent nature and a reduction in the administration of radioactive iodine (RAI) (1, 2). Compared to total thyroidectomy (TT), lobectomy poses lower rates of surgical complications, including recurrent laryngeal nerve (RLN) injury, hypoparathyroidism, bleeding, and reduced need for thyroid hormone replacement (THR) (3, 4). Despite these advantages, a recent survey of American surgeons suggested limited adoption of thyroid lobectomy for low-recurrence-risk PTCs (5).

Meanwhile, advancements in molecular testing (MT) of thyroid fine-needle aspiration (FNA) have increased the diagnostic precision of Bethesda III/IV indeterminate thyroid nodules (ITNs), thereby refining both the necessity and potentially the extent of surgical intervention. Prior to the implementation of MT for ITNs, diagnostic lobectomy rates were as high as 39% for Bethesda III and 70% for Bethesda IV nodules (6, 7), with up to two-thirds of cases yielding benign diagnoses on final pathology (6). Meanwhile, studies have reported that between 10 and 40% of patients with an ITN and a final diagnosis of thyroid cancer have an initial oncologic undertreatment with lobectomy and require completion thyroidectomy (CT) due to pathologic findings of intermediate- or high-recurrence-risk features (8, 9). This suggests that prior to MT of ITNs, there was potential overtreatment of some benign nodules and initial undertreatment of some malignant nodules, with the latter potentially requiring a second surgery for CT. MT aims to reduce unnecessary diagnostic lobectomies by either downgrading malignancy risk to permit observation or upgrading malignancy risk to direct the appropriate upfront intervention (10, 11, 12). Furthermore, studies have shown that MT is more cost-effective than diagnostic lobectomy by improving patient selection (13, 14).

Preoperative malignancy risk stratification using an integrated diagnostic pathway that included reflex MT testing of Bethesda III/IV ITNs showed an improved malignancy yield (15, 16). The primary objective of this study was to evaluate the impact of reflexive MT of ITNs on rates of lobectomy, CT, and upfront TT in a tertiary thyroid cancer setting using guideline-based thyroid lobectomy selection criteria.

## Methods

### Lobectomy criteria and molecular testing implementation

Based on the 2015 ATA guidelines (1), the Alberta thyroid tumor team, comprising all disciplines involved in the diagnosis and treatment of patients with thyroid cancer, proposed and adopted provincial lobectomy selection criteria in July 2017 as an alternative to TT for patients with low-recurrence-risk intrathyroidal thyroid cancers (Table 1) (17). In July 2020, reflexive MT was initiated for all Bethesda III/IV ITN FNAs using ThyroSPEC, a locally developed and publicly funded MT. ThyroSPEC is a mass spectrometry panel including 116 point mutations and 22 gene fusions covering 98% of single nucleotide variants and gene fusions described at least twice in thyroid cancer (COSMIC v83) (Supplemental Table 1 (see section on Supplementary materials given at the end of the article)). ThyroSPEC validation testing has been published and showed a positive predictive value (PPV) of 46–65% and a negative predictive value (NPV) of 76–91% in ITNs (15, 16). The test uses DNA and RNA extracted from residual FNA material after preparation of cytology slides. ThyroSPEC can provide the following results: insufficient sample, no mutation detected, or low, intermediate, or high risk of malignancy mutation (Supplemental Table 2). This result is provided in a report along with an estimated risk of malignancy based on local validation studies (15). ThyroSPEC results are integrated with clinical data to aid in shared decision-making with patients to decide on observation, lobectomy, or TT (16). Based on the validation studies, patients with high-risk mutations have a 93% risk of malignancy.

**Table 1:** Summary of provincial lobectomy selection criteria adapted from 2015 ATA guidelines.

Inclusion criteria	Exclusion criteria
Patient is able to provide informed consent Bethesda III/IV diagnostic lobectomy Bethesda V/VI must be < 4 cm	Personal history of head and neck radiation exposure Family history of thyroid cancer (defined as 2+ first-degree relatives) Evidence of ETE or LN involvement Multifocal cancer or contralateral high-risk nodule (US +/− FNA)

### Patient selection and study design

Patient data from July 2017 to October 2023 were retrieved from a prospective thyroid cancer database that captures patients managed by the regional thyroid cancer team. Data collection was conducted with patient consent and ethics approval in accordance with the Declaration of Helsinki and was approved by Health Research Ethics Board of Alberta (HREBA) – Cancer Committee (CC) (Ethics ID: HREBA.CC-16-0956). Inclusion criteria comprised patients with confirmed well-differentiated thyroid cancer who had undergone initial lobectomy. ThyroSPEC data from all patients with Bethesda III/IV nodules who underwent reflexive MT during the same period were also analyzed. Patients were categorized into three groups: i) Bethesda V/VI initial lobectomy, ii) Bethesda III/IV initial lobectomy without ThyroSPEC, iii) Bethesda III/IV lobectomy candidates with ThyroSPEC. Group 3 included Bethesda III/IV patients with ThyroSPEC data that had undergone initial lobectomy (group 3a) and those with Bethesda III/IV nodules that would have previously been a diagnostic lobectomy candidate but instead underwent upfront TT due to the identification of high risk of malignancy mutation(s) on ThyroSPEC concurrent with criteria favoring TT instead of lobectomy. Rates and indications for CT were compared across all groups. Due to differences in baseline characteristics between groups 2 and 3, a propensity-matched analysis was performed.

### Postoperative complications and thyroid hormone replacement

Postoperative complications were individually reviewed by a surgeon (CY) based on clinic notes and centralized medication dispensation records. Hypoparathyroidism was defined by need for postoperative replacement with a high dose of calcium with or without calcitriol, with six months serving as the cutoff between temporary and permanent hypoparathyroidism. RLN injury was identified via surgeons’ direct laryngoscopy assessment, also using six months as the cutoff between temporary and permanent injury. THR initiation post-lobectomy was documented in the database based on electronic medical record review. Preoperative thyroid-stimulating hormone (TSH) values and most recent THR doses were extracted and analyzed. The target TSH for low-recurrence-risk patients post-lobectomy was 0.5–2 mIU/L, in accordance with the 2015 ATA guidelines (1).

### Statistical analysis

All statistical analyses were performed utilizing the R statistical software package (18). Continuous variables were represented by medians and ranges, while nominal variables were expressed as frequency counts and corresponding percentages. Propensity scores were estimated using logistic regression with maximum nodule size and Bethesda category as covariates. Groups 2 and 3 were matched 1:1 using nearest-neighbor matching (MatchIt package in R). A multivariate analysis using the matched cohort was performed. The statistical significance of variables was assessed using appropriate hypothesis tests based on variable type.

## Results

The database captured 799 patients with thyroid cancer that had undergone surgery during the study period. Of them, 224 (28%) patients with a final diagnosis of well-differentiated thyroid cancer had undergone lobectomy as their initial surgery. At least 88% of patients had their initial surgery performed by a high- (25–50 cases/year) or very high-volume (>50 cases/year) thyroid surgeon. The analysis included 91 Bethesda V/VI patients (group 1), 70 Bethesda III/IV patients without ThyroSPEC data (group 2), and 48 Bethesda III/IV patients with ThyroSPEC data (group 3a) who underwent lobectomy as initial surgery. Lobectomy patients with Bethesda I/II (*n* = 15) were excluded.

During the study period, 992 ThyroSPEC tests were reflexively conducted on Bethesda III/IV FNAs, with 928 (93.5%) tests providing sufficient data. Mutations were identified in 308 FNAs, with 37 low-risk, 226 intermediate-risk, and 45 high-risk mutations. In the high-risk group, 32 patients had completed surgery during the study period: 4 lobectomies, 26 TTs, 1 CT with prior lobectomy for benign disease, and 1 aborted surgery due to unresectable disease. Of the 26 TT patients, 10 had other indications for TT, such as contralateral high-risk nodules, while 16 patients would have previously been diagnostic lobectomy candidates but, with the knowledge of the high risk of malignancy mutation combined with other clinical or radiographic features, underwent upfront TT instead (BRAF *n* = 5, TERT *n* = 3, RAS + TERT *n* = 3, BRAF + TERT *n* = 1, and ETV6/NTRK3 fusion *n* = 4). Of these 16 patients, 11 underwent appropriate upfront TT based on no longer being eligible based on therapeutic lobectomy criteria: 5 patients had nodules >4 cm (3 of which contained TERT mutations), 4 patients had nodules <4 cm but contained a TERT mutation, and 2 patients had contralateral nodules. Meanwhile, five were eligible for lobectomy but underwent TT after discussion between surgeon and patient. Four of the five lobectomy-eligible patients had ATA intermediate-risk cancers. There were four patients with high-risk mutations that were treated with initial lobectomy, three patients had ATA low-recurrence-risk cancers based on final pathology and did not require CT, while one patient underwent CT based on patient/surgeon preference.

**Figure 1:**
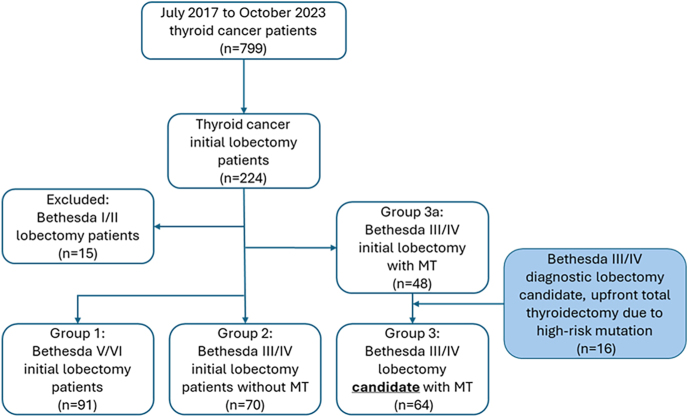
Study design – comparison of three groups of thyroid nodule patients undergoing surgery with a final diagnosis of cancer.

These 16 patients were included in group 3 as lobectomy candidates based on their Bethesda III/IV FNA (Fig. 1). Race/ethnicity data were not collected/not available in the database. Baseline preoperative characteristics were not equal between groups 2 and 3 with regard to maximum nodule size on ultrasound and Bethesda III/IV category (Table 2). Groups 2 and 3 underwent a 1:1 propensity score matching based on nodule size and Bethesda category to account for these differences.

**Table 2:** Baseline preoperative patient demographics in the three groups – Group 1: Bethesda V/VI lobectomy patients; Group 2: Bethesda III/IV no ThyroSPEC; Group 3: Bethesda III/IV with ThyroSPEC. Data are presented as median (IQR) or as *n* (%). Statistically significant ( *P<*0.05) values are presented in bold.

	Group 1	Group 2	Group 3	*P* value*
*n*	91	70	64	
Age at diagnosis	45 (36–55)	47 (39–59)	48 (38–59)	0.887
Sex				1.000
Female	67 (74%)	56 (80%)	51 (80%)	
Male	24 (26%)	14 (20%)	13 (20%)	
History of radiation exposure				0.335
No	88 (97%)	64 (91%)	62 (97%)	
Yes	0	2 (3%)	1 (1.5%)	
Unknown	3 (3%)	4 (6%)	1 (1.5%)	
Family history of thyroid cancer				0.214
No	87 (96%)	63 (90%)	62 (97%)	
Yes	0	0	0	
Unknown	4 (4%)	7 (10%)	2 (3%)	
Maximum US nodule size (cm)	1.5 (1.1–2)	3.0 (2.3–4.3)	2.5 (1.6–3.8)	**0.019**
Bethesda category				**<0.001**
Bethesda III	n/a	30 (43%)	49 (77%)	
Bethesda IV	n/a	40 (57%)	15 (23%)	
Bethesda V	17 (19%)	n/a	n/a	
Bethesda VI	74 (81%)	n/a	n/a	

* Group 2 vs Group 3.

### Completion thyroidectomy rates and indications

**Figure 2:**
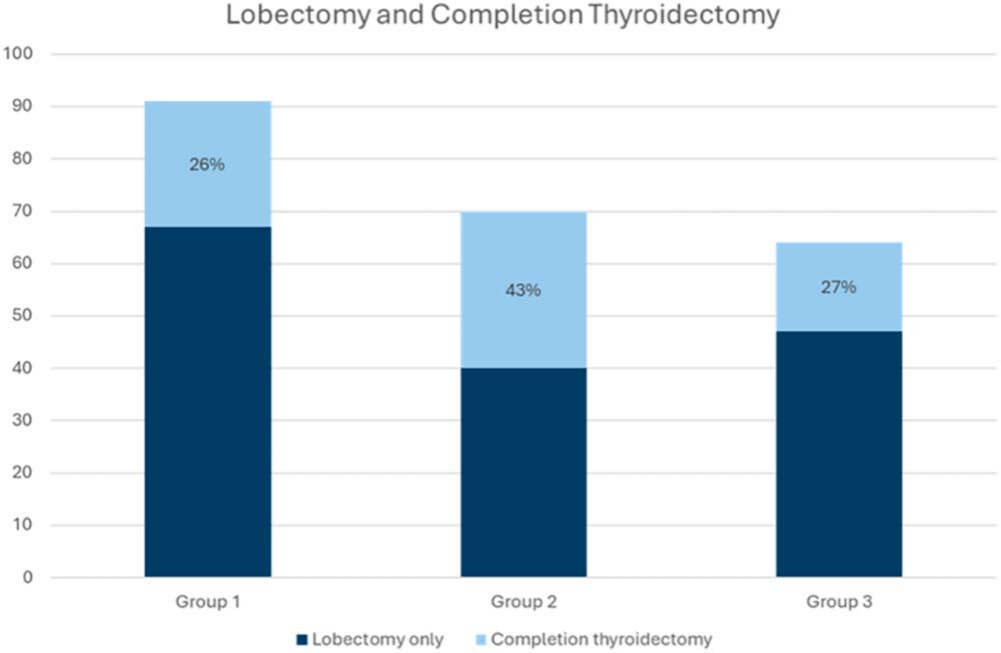
Lobectomy and completion thyroidectomy rates by group. Group 1: Bethesda V/VI initial lobectomy; Group 2: Bethesda III/IV initial lobectomy without MT; Group 3: Bethesda III/IV lobectomy candidates with MT.

There was 96–100% compliance with various preoperative lobectomy selection criteria (Supplemental Table 3). A total of 71 initial lobectomy patients underwent CT, with CT rates of 26, 43, and 27% in each respective group (*P* = 0.049, Fig. 2). Twenty-one patients had more than one indication for CT. Vascular invasion (VI) was a common indication across all groups, occurring at a rate of 11–20%. Tumor size >4 cm was a frequent indication for CT in groups 2 and 3 (17 and 13%, respectively). It was the sole indication for CT in eight patients, including three in group 3 – none of whom had high-risk molecular mutations. Tumor size >4 cm was not an indication for CT in group 1 as these patients had a preoperative malignant diagnosis and thus were excluded from lobectomy based on local criteria and underwent upfront TT. Aggressive histology was an indication for CT in 5–10%, while gross extrathyroidal extension (ETE) and lymph node (LN) involvement were less common indications (≤5%) as these findings typically resulted in upfront or intraoperative conversion to TT. Microscopic ETE or low-volume microscopic LN (<5 LN, <0.5 cm) involvement without extranodal extension was not included as reasons for CT. Other less common indications for CT were contralateral nodule features or patient preference. Included in the CT dataset are four patients that underwent CT more than one year after initial lobectomy: one due to new LN metastasis, one due to contralateral nodule growth, one due to a new contralateral Bethesda V nodule, and one due to patient preference (Table 3). RAI was administered to 51 of 71 (72%) CT patients. The difference in CT rates was maintained in the propensity-matched cohort with 41% in group 2 and 21% in group 3 (*P* = 0.049).

**Table 3:** Rates and indications for completion thyroidectomy in the three groups – Group 1: Bethesda V/VI lobectomy patients; Group 2: Bethesda III/IV no ThyroSPEC; Group 3: Bethesda III/IV with ThyroSPEC. It is to be noted that 21 patients had >1 indication for CT. Data are presented as *n* (%).

	Group 1	Group 2	Group 3
	(*n* = 91)	(*n* = 70)	(*n* = 64)
Completion thyroidectomy	24 (26%)*	30 (43%)*	17 (27%)*
Vascular invasion	13 (14%)	14 (20%)	7 (11%)
Tumor size > 4 cm	0	12 (17%)	8 (13%)
Aggressive histology	10 (10%)	4 (6%)	3 (5%)
PTC tall cell	8	3	0
PTC solid variant	1	1	2
PTC high grade	1	0	1
ETE	5^†^ (5%)	2^§^ (3%)	1^║^ (2%)
LN metastasis	3^‡^ (3%)	0	0
Contralateral nodules	0	3 (4%)	1 (2%)
Patient preference	0	2 (3%)	1 (2%)

PTC, papillary thyroid cancer; ETE, extrathyroidal extension; LN, lymph node.

* *P* < 0.05.

^†^ Five strap muscle gross ETE deferred to pathology fibrosis vs invasion.

^‡^ One >3 cm LN-positive; 1 > 5 cm LN-positive; 1 new LN metastasis identified on follow-up.

^§^ One RLN gross ETE intentionally staged to defer to final pathology; 1 positive margin with micro-ETE.

^║^ One strap muscle gross ETE deferred to pathology fibrosis vs invasion.

### Impact of reflexive ThyroSPEC testing on Bethesda III/IV nodules

After the introduction of reflexive ThyroSPEC testing, there was an increase in upfront TT from 33 to 46% (*P* = 0.015) among patients with Bethesda III/IV nodules. Sixteen patients with Bethesda III/IV nodules that would have previously undergone diagnostic lobectomy as initial surgery instead received upfront TT after confirmation of malignancy through detection of a high-risk mutation. Of these 16 patients, 11 underwent appropriate upfront TT based on no longer being eligible based on therapeutic lobectomy criteria. Meanwhile, five were eligible for lobectomy but underwent TT after discussion between surgeon and patient. Four of the five lobectomy-eligible patients had ATA intermediate-risk cancers and would have required CT had they undergone lobectomy initially. There were four Bethesda III/IV patients with high-risk mutations that underwent initial lobectomy, of whom only one underwent CT due to patient/surgeon preference. In a multivariable logistic regression model after adjusting for age at diagnosis, maximum nodule size, and Bethesda category, the use of ThyroSPEC was independently associated with a significantly lower likelihood of CT compared with no ThyroSPEC testing (OR = 0.35, 95% CI: 0.12–0.95; *P* = 0.046; Table 4).

**Table 4:** Multivariable logistic regression model predicting completion thyroidectomy in groups 2 and 3 after propensity score matching.

Covariate (reference)	Coefficient ± SE	OR (95% CI)	*P* value
ThyroSPEC yes (vs no)	−1.06 ± 0.53	0.35 (0.12, 0.95)	0.046
Age at diagnosis	−0.02 ± 0.02	0.98 (0.94, 1.01)	0.186
Maximum nodule size	0.15 ± 0.18	1.17 (0.82, 1.67)	0.390
Bethesda category IV (vs III)	0.07 ± 0.59	1.07 (0.32, 3.39)	0.907

### Postoperative complications and thyroid hormone replacement

The surgical complication rate for lobectomy was low (2%); four patients experienced temporary RLN injury, and one patient required intentional RLN sacrifice due to tumor involvement. In the patient with intentional RLN sacrifice, thyroidectomy was undertaken in a staged fashion as initial cytologic diagnosis was indeterminate. CT carried a higher rate of complication (7%) mainly due to temporary hypoparathyroidism (4%) (Table 5).

**Table 5:** Surgical complications and thyroid hormone replacement rates.

Complication	Initial lobectomy (*n* = 209)	Completion thyroidectomy (*n* = 71)
Temporary RLN injury	4 (2%)	1 (1%)
Permanent RLN injury	1* (0.5%)	0 (0%)
Temporary hypoparathyroidism	0 (0%)	3 (4%)
Permanent hypoparathyroidism	0 (0%)	1 (1%)
Hormone replacement		100%
Lobectomy-only patients	44%	
If pre-op TSH > 2	77%	

* One intentional RLN sacrifice due to tumor involvement – intentionally staged surgery due to RLN involvement and indeterminate cytologic diagnosis.

Postoperatively, 44% of lobectomy-only patients required initiation of THR, with a median time to initiation of 4 months (IQR: 2–8 months). Patients on THR prior to lobectomy surgery were excluded. The final median levothyroxine dose was 88 μg (IQR: 75–100 μg) to target a TSH of 0.5–2 mIU/L. Notably, 77% of patients with preoperative TSH > 2 mIU/L required THR postoperatively. Fisher’s exact test showed a statistically significant association between preoperative TSH values and postoperative THR requirements (*P* < 0.001), with regression analysis showing an odds ratio of 1.88 for THR when preoperative TSH > 2 mIU/L. Data were not available to evaluate the impact of chronic lymphocytic thyroiditis on the need for THR post-lobectomy.

## Discussion

Our study demonstrates that the implementation of MT for ITNs significantly improved surgical decision-making. We observed a decrease in CT rates for Bethesda III/IV patients post-ThyroSPEC implementation and a corresponding increase in appropriate upfront TT. These findings were maintained when groups were matched for size and Bethesda category, and ThyroSPEC remained independently associated with a significantly lower likelihood of CT in multivariate analysis. These results underscore the value of MT in optimizing surgical strategies and minimizing the need for two-stage surgeries. This aligns with a recent study looking at reflexive MT for all indeterminate FNAs, which found a 14% increased malignancy diagnostic yield in patients selected for surgery (15).

Connelly *et al.* have recently compared three commercially available molecular tests (ThyroSeq, ThyGeNEXT/ThyraMIR, and Afirma) in ITNs and identified PPVs between 44 and 67% and NPVs between 66 and 80% (19), which is very comparable with ThyroSPEC (PPV: 46–65%; NPV: 76–91%) (15, 16). While ThyroSPEC includes 98% of single nucleotide variants and gene fusions described at least twice in thyroid cancer (COSMIC v83) and detects the most prevalent high-risk mutations used in ThyroSeq v3 (20), ThyroSPEC does not detect mutations reported with low prevalence and outside of hotspot regions. It is possible that rare high-risk mutations were missed by ThyroSPEC; however, given the low incidence of these mutations, this likely has a minimal impact on CT rates.

### Completion thyroidectomy indications

CT is undertaken for many reasons, including enabling RAI administration and facilitating thyroglobulin monitoring (1). However, limiting unnecessary CT is important to reduce patient burden, such as time off work, prolonged recovery, psychological impact, and healthcare costs (21, 22, 23). The CT rates in this study for groups 1 and 2 are comparable to recent reports for therapeutic (9, 24) and diagnostic lobectomies (25, 26, 27), respectively. It is important to reiterate that the present study utilized data from a cancer database; thus, only diagnostic lobectomies with a final malignant diagnosis were included, significantly overestimating the CT rate among all ITNs.

The observed decline in CT rates following ThyroSPEC implementation reflects enhanced preoperative risk assessment. When MT confirms malignancy in ITNs, surgeons can more confidently evaluate other preoperative features, such as tumor size, contralateral lobe nodules, and patient preference, in the context of a known malignancy. Patients with malignant mutations and concerning features can proceed directly to upfront TT, while those without high-risk malignant mutations or other high-risk clinical or radiographic features can be considered for lobectomy.

Vascular invasion and aggressive histology were both common indications for CT across all groups. Both features raise the risk of recurrence prompting CT but cannot be determined based on preoperative evaluation. Tumor size >4 cm was an indication for upfront TT in patients with Bethesda V/VI nodules or Bethesda III/IV nodules with a high-risk mutation. An ITN >4 cm is not considered an indication for upfront TT but remained a common CT indication in groups 2 and 3. While tumor size >4 cm is associated with an increased risk of ETE, LN metastasis, and distant metastasis (28), evidence regarding tumor size as an independent negative prognostic factor remains inconsistent (29, 30). Considering this, the role of tumor size >4 cm as a stand-alone trigger for CT should be carefully evaluated, especially in the absence of other high-risk features.

### Postoperative complications and thyroid hormone replacement

The benefits of lobectomy over TT are the lower surgical complication rates (31), lower likelihood of requiring THR (50–80% vs 100%) (32, 33, 34), improved quality of life (3), and cost-effectiveness (22, 23). In this study, the lobectomy procedure had very low complication rates (2%), while CT had a higher complication rate, with 4% temporary and 1% permanent hypoparathyroidism and 1% temporary RLN injury. These complication rates are comparable to those of a recent meta-analysis (31). It is important to note that these surgeries were done by high-volume thyroid surgeons. TT complications were not assessed in this study. Studies have shown that CT has similar or lower complication rates than TT (35, 36, 37), although selection bias may exist with CT patients being sent to high-volume surgeons. Based on the 2015 ATA guidelines’ TSH target of 0.5–2 mIU/L for low-risk thyroid cancers, 44% of lobectomy patients required THR initiation. The 2025 ATA guidelines have changed this recommendation to a target TSH within normal reference range, which will likely decrease the need for THR initiation in lobectomy patients (38).

### Limitations

There are several limitations to this study. First, we did not directly assess long-term follow-up data or recurrence. As a surrogate marker for recurrence, we observed only four patients that required CT more than one year after initial lobectomy. Data on long-term lobectomy follow-up have been well established elsewhere (39, 40). Second, while we were able to review CT rates, we do not know the rate of intraoperative conversion from lobectomy to TT in this cohort. This is another factor that needs to be discussed with patients during surgical consent, as intraoperative conversion rates can be around 20% (9, 24). Third, this study only included patients with malignant histology and does not capture patients with ITNs with final benign histology. This design is expected to overestimate rates of CT among Bethesda III/IV patients; however, it serves to answer this study’s main question which is to compare ITNs with and without reflexive MT while applying the same lobectomy inclusion/exclusion criteria. Finally, there may also have been lobectomy candidates that chose surveillance or upfront TT due to patient preference that are not captured in this study.

The cost-effectiveness of MT could be improved by only testing patients that do not have other indications for TT, such as contralateral high-risk or symptomatic nodules. However, this process would require clinical assessment by a thyroid specialist to occur between initial thyroid FNA and MT (as opposed to the current method of reflexive testing of ITNs) and may necessitate obtaining a second biopsy sample or result in delays to care. In the public healthcare system, wait times for patients with ITNs to consult with a thyroid specialist can be considerable, and the identification of a high-risk mutation will appropriately expedite the consultation. Further health economic studies for reflexive MT of ITNs will need to be explored.

## Conclusion

In summary, appropriate patient selection for lobectomy remains challenging and there is need for careful discussion of risks versus benefits, likelihood of CT, and need for THR when consenting a patient for thyroid surgery. The implementation of MT for ITNs significantly reduced CT rates while increasing appropriate upfront TT. Indications for CT were most often due to pathology findings, such as VI or aggressive histological subtypes, which cannot be detected preoperatively based on FNA cytology alone. Appropriate upfront surgical decision-making supported by MT has the potential to decrease patient burden in terms of anxiety, time off work, need for recurrent surgery, and the cost to the healthcare system.

## Supplementary materials



## Declaration of interest

ME and RP receive licensing fees for ThyroSPEC. All other authors have no disclosures.

## Funding

Initial ThyroSPEC grant funding was provided by the Thyroid Foundation of Canada, Genome Alberta, and the Alberta Cancer Foundation. MT costs post-implementation of ThyroSPEC were borne by Alberta Precision Laboratories (a crown corporation). No direct funding was received for this study.

## Author contribution statement

CTY: conceptualization (lead), data curation and analysis (equal), data interpretation (lead), writing (lead), review and editing (lead). JW: conceptualization (supporting), data curation and analysis (equal), writing (supporting), review and editing (supporting). SG: conceptualization (supporting), review and editing (equal). PS: review and approval (equal). SA: data curation (supporting). AB: review and approval (equal). ME: review and approval (equal). RP: conceptualization (supporting), supervision, review and editing (equal).
