# Supplementary material for: Impact of reflexive preoperative molecular testing for indeterminate nodules on lobectomy and completion thyroidectomy rates
Source: Eur Thyroid J. 2026 Mar 13;15(2):ETJ250189. doi: 10.1530/ETJ-25-0189 (PMC13015782; doi:10.1530/ETJ-25-0189)
Supplement: Supplementary file 1 [file supplementary_materials.pdf]

Supplemental Table 1. List of molecular mutations/fusions tested with ThyroSPEC (as of February 2024)

Genes (number of mutations)	Fusions (number of fusions)
AKT1 (1)	*AGK::BRAF* (1)
BRAF (7)	*AKAP::BRAF* (1)
CTNNB1 (11)	*CRTC1::MAML2* (1)
DICER1 (3)	*EML4::ALK* (1)
EGFR (1)	*ETV6::NTRK3* (1)
EIF1AX (4)	*IGF2BP3::THADA* (1)
EZH1 (1)	*PAX8::PPARG* (3)
HRAS (15)	*RET::PTC* (4)
IDH1 (1)	*RPS2P32::THADA* (1)
KRAS (17)	*SND1::BRAF* (1)
NRAS (15)	*SQSTM1::NTRK3* (1)
PIK3CA (5)	*STRN::ALK* (1)
PTEN (1)	*TFG::NTRK1* (1)
RET (19)	*TMEM233::PRKAB1* (1)
SPOP (1)	*TPM3::NTRK1* (1)
TERT (2)	*TPR::NTRK1* (1)
TP53 (10)	
TSHR (2)	

Supplemental Table 2. Molecular mutations/fusions according to risk of malignancy category

**Risk category**	**Mutation/Fusion**
Low-risk	TSHR, EZH1, SPOP, PTEN
Intermediate-risk	NRAS, HRAS, KRAS, BRAFK601E, EIF1AX, IDH1, DICER1, TP53 or rearrangements in PPARG, THADA
High-risk	BRAFV600E, TERT, or rearrangements in BRAF, RET, NTRK1, NTRK3, BRAF + TERT, RAS + TERT, RAS + EIF1AX, AKT1, PIK3CA, CTNNB1, EGFR, or rearrangements in ALK

Supplemental Table 3. Adherence to preoperative lobectomy selection criteria

Criteria	Adherence Rate
No personal history of head and neck radiation	97.8%
No family history of thyroid cancer	100%
If Bethesda V/VI, must be <4cm	100%
Evidence of gross ETE or LN involvement	96.9%[a], 99.6% respectively
No US detectable multifocal cancer	99.6%
Contralateral lobe is free of high-risk nodule(s) (US +/- FNA)	99.6%

[a]3 cases with RLN involvement intentional sacrificed or shaved off RLN. TT deferred to final pathology as cytology indeterminate. 1 shaved off RLN, TT deferred to assess nerve; 3 unsure if strap invaded versus fibrosis – deferred to pathology
